# Gemcitabine-Induced Thrombotic Microangiopathy Managed Conservatively in a Patient of Breast Cancer

**DOI:** 10.7759/cureus.28433

**Published:** 2022-08-26

**Authors:** Ghulam Mujtaba Ghumman, Huda Fatima, Tyler L Johnston, Rachel Leis, Vinod Khatri

**Affiliations:** 1 Internal Medicine, St. Vincent Mercy Medical Center, Toledo, USA; 2 Emergency Medicine, St. Vincent Mercy Medical Center, Toledo, USA; 3 Pharmacology, St. Vincent Mercy Medical Center, Toledo, USA; 4 Critical Care Medicine, St. Vincent Mercy Medical Center, Toledo, USA

**Keywords:** creatinine, thrombocytopenia, anemia, thrombotic microangiopathy, chemotherapy, gemcitabine

## Abstract

Thrombotic microangiopathy (TMA) consists of a group of occlusive microvascular disorders, which include thrombotic thrombocytopenic purpura (TTP) and hemolytic uremic syndrome (HUS). TMA can be classified as primary or secondary based on the etiology. Gemcitabine-induced TMA is a rare side effect of the drug with varying clinical presentations. We present a case involving the classic triad of microangiopathic hemolytic anemia (MAHA), thrombocytopenia, and renal failure associated with gemcitabine. Gemcitabine was immediately stopped, and our patient's condition improved with conservative management.

## Introduction

Thrombotic microangiopathy (TMA) comprises clinical disorders of thrombotic thrombocytopenic purpura (TTP) and hemolytic uremic syndrome (HUS). TMA is characterized by systemic or intra-renal platelet aggregation with thrombocytopenia, microangiopathic hemolytic anemia (MAHA), and end-organ damage [1]. TMA can be classified as primary versus secondary based on etiology. Chemotherapy regimens can be a secondary cause of TMA; however, TMA induced by the chemotherapeutic drug gemcitabine has been rarely reported, with only a few reported cases in the literature so far [2]. We present a case of gemcitabine-induced TMA, which improved with conservative management.

## Case presentation

A 66-year-old female with a past medical history of stage IV breast cancer presented to the emergency department (ER) for the evaluation of acute-onset shortness of breath. The patient had been on gemcitabine therapy for the past four months, with the last dose received on the day of the presentation. The patient had initially presented to the chemotherapy infusion center for her routine session of gemcitabine chemotherapy. After receiving chemotherapy, she started having shortness of breath, gradually worsening with associated orthopnea. On arrival at ER, the patient was in acute respiratory distress with a respiratory rate of 29 breaths per minute, blood pressure of 181/106 mmHg, and heart rate of 96 beats per minute. Physical examination showed bilateral rhonchi on chest examination and lower extremity pitting edema. No jugular venous distention was appreciated. Chest imaging showed evidence of pulmonary edema and bilateral pleural effusions. The patient was placed on noninvasive ventilation with improvement in respiratory status over the next couple of days. Blood workup showed anemia, thrombocytopenia, elevated creatinine, and evidence of hemolysis with elevated serum lactate dehydrogenase (LDH) and low haptoglobin (Table 1).

**Table 1:** Laboratory values with reference range - indicates that the laboratory value was not checked

Parameter (units)	Patient value				Reference range
	On admission	Nadir during hospitalization	At discharge	At the 6-month follow-up	
Hemoglobin (g/dL)	7.1	6.3	8.1	8.5	11.9–15.1
Platelets (k/uL)	115	17	102	149	138–453
Creatinine (mg/dL)	2.99	4.30	2.60	1.41	0.50–0.90
Blood urea nitrogen (mg/dL)	70	124	26	22	8–23
Lactate dehydrogenase (U/L)	1640	1640	800	300	135–214
Haptoglobin (mg/dL)	<10	<10	-	35	30–200
ADAMTS13 (%)	>50	-	-	-	64–134
Urine protein (mg/dL)	64	-	-	-	0–14
Urine creatinine (mg/dL)	18.6	-	-	-	0.5–1.1
Proteinuria (g/day)	3.4	-	-	-	0.15
Serum albumin (g/dL)	3.6	-	-	-	3.4–5.4

**Figure 1:**
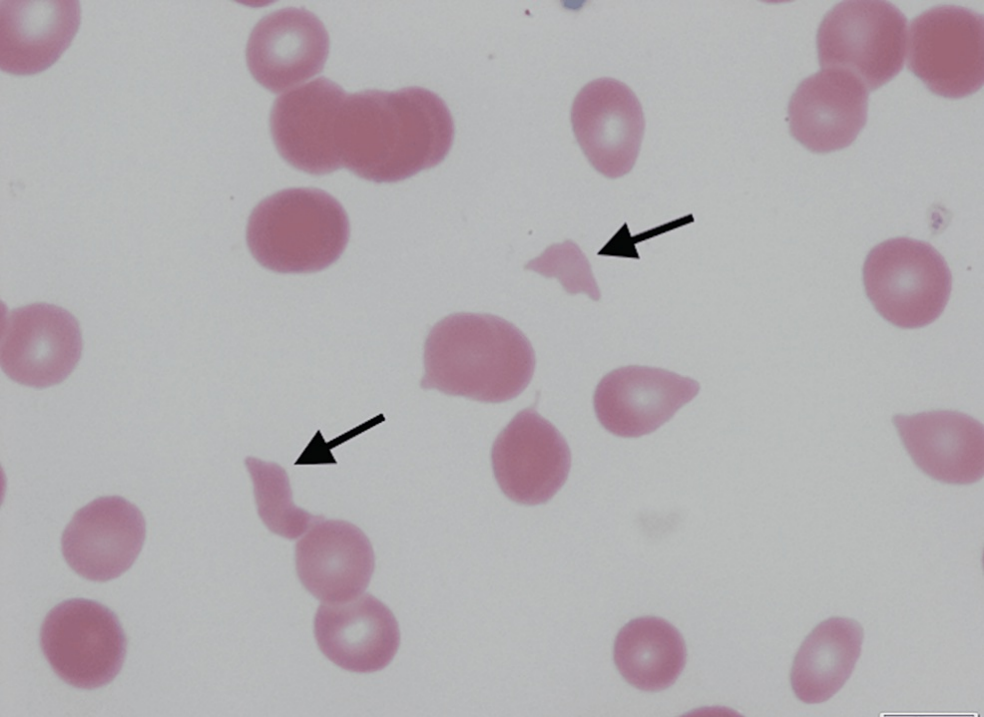
Peripheral blood smear showing schistocytes (arrows)

A peripheral blood smear showed schistocytes (Figure 1).

**Figure 2:**
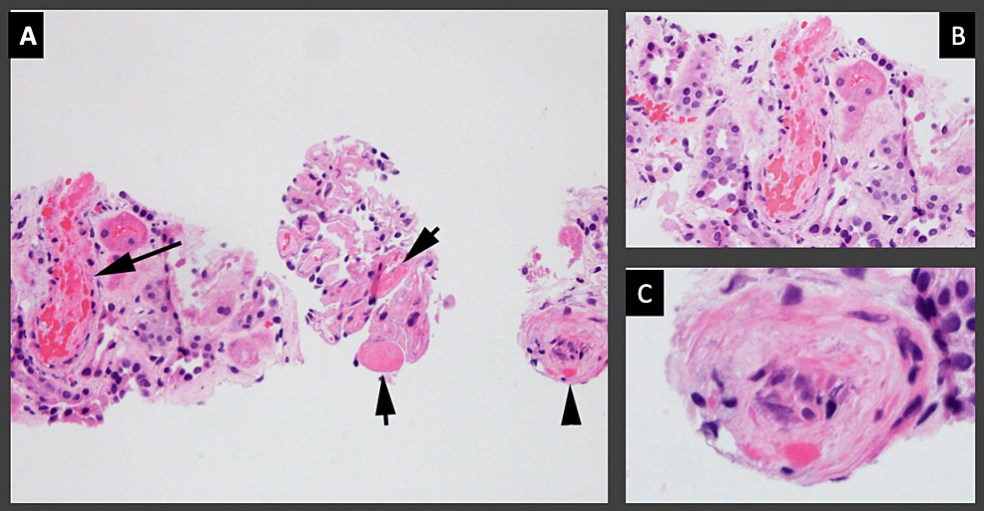
Renal biopsy showing features of thrombotic microangiopathy (A) Acutely injured arteriole with occlusion of the lumen by fibrin and platelets entrapping and fragmenting the RBCs with the formation of schistocytes (large arrow) and capillaries filled with fibrin (short arrow), while another arteriole is demonstrating a more chronic phase with the lumen containing swollen endothelial cells and macrophages (arrowhead). (B) Higher power view of the acutely injured arteriole, shown in A with the large arrow. (C) Higher power view of the second arteriole, shown in A with the arrowhead

The Coombs test was negative. The platelets and hemoglobin continued to drop during hospitalization. Due to concerns regarding TTP, plasmapheresis was started, which was then stopped (after two sessions) after ADAMTS13 showed a value >50%, which excluded the diagnosis of TTP. Due to a persistent rise in creatinine and blood urea nitrogen (BUN) with oliguria, the patient was started on hemodialysis with nephrology recommendations. Renal ultrasound was unremarkable with normal renal cortical echogenicity. Complement levels were normal. ANA and hepatitis B and C were negative. Transthoracic echocardiogram showed an ejection fraction of 45% with grade-I diastolic dysfunction. The patient received packed red cells and platelet transfusions to improve the hemoglobin and platelet count. A left renal biopsy was carried out, which showed thrombotic microangiopathy, mild tubular injury superimposed on diffuse mild to moderate tubular atrophy, and interstitial fibrosis (Figure 2).

The patient’s clinical condition continued to improve during hospitalization. She was on room air at discharge but still dependent on hemodialysis. Gemcitabine was stopped on discharge. Six months after discharge, the patient was off hemodialysis, and her thrombocytopenia had improved.

## Discussion

TMA is classified as primary when the etiology is idiopathic and secondary when the constellation of symptoms occurs secondary to an infection, autoimmune disorders, pregnancy, malignancy, drugs, bone marrow transplantation, or cytotoxic chemotherapy. TMA can be secondary to cancer itself, but that is more likely to present as TTP, while TMA secondary to chemotherapy manifests as symptoms of HUS [3]. HUS is associated with a triad of anemia, thrombocytopenia, and renal dysfunction (predominantly), while TTP commonly manifests as a pentad of thrombocytopenia, MAHA, renal failure, neurological abnormalities, and fever. Each of these can have further subtypes based on different mechanisms. For instance, HUS can be further classified as typical HUS involving diarrhea with Shiga toxin as a presenting symptom and atypical HUS which lacks diarrhea [4]. Among the chemotherapy regimens, the one involving gemcitabine is a rarely reported cause of TMA. It is a pyrimidine antimetabolite used, both as a single agent and in combination, in the treatment of pancreatic, pulmonary, urothelial, breast carcinoma, and lymphoma [5]. The mechanisms for drug-induced TMA can include immune-mediated reactions and drug-related toxicity. The presence of circulating antibodies and improvement with immunotherapy drugs in some cases point to an immune-mediated mechanism, although no clear mechanism has been identified with respect to gemcitabine [6].

The clinical manifestations of gemcitabine-induced TMA can include dyspnea related to pulmonary edema, peripheral edema, hematuria, neurological abnormalities with laboratory evidence of worsening anemia, and thrombocytopenia with elevated markers of hemolysis [7]. According to the literature, the incidence of gemcitabine-induced TMA is around 0.015% (range: 0.008-0.078%) [7]; however, the actual incidence may be underestimated due to poor recognition of the cause and failure to diagnose mild symptoms of HUS. While most cases have reported the use of gemcitabine as a single agent, the risk of gemcitabine-associated TMA appears to increase with cumulative dosing as well as with the use of other drugs as a part of combination therapy [8]. However, our patient was only on gemcitabine. While malignancy can be a cause of HUS, this was not the case with our patient as she did not have any detectable disease recurrence. Moreover, her ADAMTS13 level was greater than 50%, effectively ruling out TTP as an alternative diagnosis.

The mainstay of treatment includes cessation of gemcitabine and supportive management, which includes blood and platelet transfusions, dialysis, and control of respiratory symptoms [8]. While the role of plasma exchange therapy is well established in TTP, its role in chemotherapy-related TMA is unclear, and there is no convincing data to support the use of plasmapheresis in these patients. Nonetheless, plasma exchange therapy should be started in all suspected cases (until the final diagnosis is reached) as the features of TTP and HUS overlap and because the establishment of diagnosis takes time [9]. Rituximab has also been utilized as a treatment option, and two patients with refractory gemcitabine-induced TMA reportedly had a successful response to rituximab [10,11]. Eculizumab has also been used in some cases of gemcitabine-induced TMA with reported clinical improvement [12]. Our patient was managed conservatively. The prognosis of the HUS related to malignancy and chemotherapy is poor, with mortality rates of around 50%. Studies have shown that nearly 70% of patients require dialysis in the acute phase, while a significant proportion (26%) end up on long-term dialysis [13,14].

## Conclusions

TMA and its subtypes can be potentially fatal. Gemcitabine has been identified as one of the potential causes of secondary TMA presenting as HUS. It is important to be vigilant for signs and symptoms of TMA in patients on gemcitabine therapy and to stop it immediately if symptoms of this disorder develop. The prognosis of HUS related to chemotherapy and malignancy is poor, with high mortality rates. The mainstay of treatment is the cessation of gemcitabine and supportive management, while various novel treatment options have been reported in the literature.
